# A retrospective single-center analysis of prenatal diagnosis and follow-up of 626 chinese patients with positive non-invasive prenatal screening results

**DOI:** 10.3389/fgene.2022.965106

**Published:** 2022-09-19

**Authors:** Xiufen Bu, Shihao Zhou, Xu Li, Shihong Li, Hongyu Li, Siyi Ding, Jun He, Siyuan Linpeng

**Affiliations:** ^1^ Department of Genetics and Eugenics, Changsha Hospital for Maternal & Child Health Care Affiliated to Hunan Normal University, Changsha, China; ^2^ Department of Basic Medicine, Yiyang Medical College, Yiyang, China

**Keywords:** karyotyping, positive predictive value, invasive prenatal diagnosis, chromosomal microarray analysis, non-invasive prenatal screening

## Abstract

This study explored the diagnostic efficiency of different prenatal diagnostic approaches for women with positive non-invasive prenatal screening (NIPS) results by analyzing their clinical information and pregnancy outcomes. We collected data on 626 NIPS-positive pregnant women from January 2017 to June 2021 and arranged subsequent prenatal diagnostic operations for them after genetic counseling, along with long-term intensive follow-up. A total of 567 women accepted invasive prenatal diagnosis (IPD) (90.58%), and 262 cases were confirmed as true positives for NIPS. The positive predictive values for trisomies 21 (T21), 18 (T18), and 13 (T13); sex chromosome aneuploidies (SCAs); rare autosomal trisomies (RATs); and microdeletion and microduplication syndromes (MMS) were 81.13%, 37.93%, 18.42%, 48.83%, 18.37%, and 41.67%, respectively. Discordant results between NIPS and IPD were observed in 48 cases, with the discordance rate being 8.47%. Additionally, there were 43 cases with discordant results between karyotyping and chromosomal microarray analysis (CMA)/copy number variation sequencing. Additional reporting of RATs and MMS with routine NIPS that only detects T21/T18/T13 and SCAs can yield more accurate diagnoses. However, NIPS cannot be used as a substitute for IPD owing to its high false positive rate and discordance with other diagnostic methods. Therefore, we recommend CMA combined with karyotyping as the preferred method for accurately diagnosing NIPS-positive women.

## Introduction

Approximately 900,000 new cases of congenital disabilities, including congenital structural, functional, and/or biochemical-molecular defects, are recorded yearly in China, with a prevalence rate of approximately 56.0 per 1,000 live births (Dai et al., 2011). Approximately 80% of congenital disability cases have unknown causes; however, strong evidence suggests that genetic conditions contribute to their etiologies (Feldkamp et al., 2017; Sun et al., 2018). Chromosomal abnormalities such as trisomies 21 (T21), 18 (T18), and 13 (T13) and sex chromosome aneuploidies (SCAs) are the main causes of congenital disabilities (Xie et al., 2021). In addition, multiple lines of evidence indicate that copy number variants (CNVs) in submicroscopic chromosomal structures can also play an important role in the etiology of some congenital disability cases (Lupo et al., 2019) (Hobbs et al., 2014). Congenitally disabled infants with chromosomal or genetic abnormalities are often diagnosed with varying degrees of intellectual disabilities, multiple malformation syndrome, growth retardation, and multiple dysfunction syndrome (Jackson et al., 2020), resulting in a considerable economic burden for families and society, thus highlighting the importance of prenatal genetic screening and diagnosis.

Non-invasive prenatal screening (NIPS), introduced into clinical practice in 2012, has gained popularity in recent years as a screening test for genetic abnormalities during pregnancy. NIPS identifies genetic abnormalities by analyzing maternal blood during pregnancy by employing next-generation sequencing (NGS) techniques to detect highly fragmented circulating cell-free fetal DNA (cffDNA), which is derived from placental tissues and has rapid post-delivery clearance profiles (Breveglieri et al., 2019; Chiu and Lo, 2021). Therefore, the risks associated with conventional invasive techniques are avoided, making it more acceptable to pregnant women as a preferred diagnostic method than conventional methods. In the last 10 years, numerous studies have focused on the clinical applicability of NIPS, mostly for detecting common autosomal trisomies (T21, T18, and T13) and SCAs (Bedei et al., 2021). Nevertheless, with the rapid development of NGS technologies, such as whole genome sequencing, the applicability of NIPS has been gradually extended to rare autosomal trisomies (RATs) and microdeletion and microduplication syndromes (MMS). NIPS involves the direct examination of DNA derived from the placenta, which has the same origin as the fetus, and has been shown to have much higher specificity and sensitivity than that of traditional serum analyte screening, which requires considering additional biochemical indicators as well as maternal age, race, and weight (D’ambrosio et al., 2019). However, NIPS-based identification of enhanced risk is susceptible to false positives; therefore, invasive prenatal diagnostic approaches such as amniocentesis, chorionic villi sampling, and/or percutaneous umbilical cord blood sampling are recommended to identify false positive findings (Liang et al., 2018; La Verde and De Falco, 2021).

In this study, we present the clinical data of 626 NIPS-positive cases detected based on whole genome sequencing of patients at a tertiary medical center in China from January 2017 to June 2021. The confirmatory invasive test results and detailed follow-up information were summarized to assess the performance of NIPS in detecting common autosomal trisomies, SCAs, RATs, and MMS and to analyze the clinical outcomes following high-risk results. In addition, we analyzed and compared the invasive test results with those of karyotyping and chromosomal microarray analysis (CMA)/copy number variation sequencing (CNV-seq) to evaluate the accuracy, efficacy, and incremental yield of CMA/CNV-seq compared with those of karyotyping for routine prenatal diagnosis.

## Materials and methods

### Ethics approval and consent to participate

The study design and protocol were reviewed and approved by the ethics committee of Changsha Hospital for Maternal and Child Health Care (No. 2021004). All methods and clinical procedures were performed in accordance with the relevant guidelines and regulations. All pregnant women received genetic counseling and provided informed consent before testing.

### Subjects

From January 2017 to June 2021, 53,437 pregnant women underwent NIPS at our hospital, and 626 received positive results. The average age of the pregnant women who received positive NIPS results was 31.0 ± 5.7 years. Among the study participants, 122 women were of advanced maternal age (≥35 years), accounting for 19.49% of the study population. Maternal blood was collected at gestational ages approximately between 12 and 28 weeks. Table 1 lists the demographic characteristics of these women.

**TABLE 1:** Demographics of the 626 women with NIPS positive results.

Characteristics	*N*	Constituent ratio (%)
Gestational age at NIPS (weeks)		
First trimester (6–13 weeks)	10	1.60
Second trimester (14–27 weeks)	615	98.24
Third trimester (≥28 weeks)	1	0.16
Maternal age (years)		
<30 years	287	45.85
30–34 years	217	34.66
35–39 years	87	13.90
≥40years	35	5.59
Pregnancy		
Singleton pregnancy	623	99.52
Twin pregnancy	3	0.48
Pregnancy method		
Natural conception	611	97.60
Assisted reproductive conception	15	2.40

On receiving a positive NIPS result, the pregnant women received prenatal genetic consultation with a professional geneticist and were informed of the importance of prenatal diagnosis to ascertain the true positives identified by NIPS. In our research, 567 women accepted the prenatal genetic diagnosis, while 59 women refused. The prenatal genetic diagnosis was carried out according to our routine experimental method and was completed at our prenatal diagnosis center. Five hundred and sixty-five women underwent amniocentesis at a suitable gestational stage (16–28 weeks), while two underwent percutaneous umbilical cord blood sampling (>28 weeks).

### Non-invasive prenatal screening

We collected 5 ml of peripheral blood from the pregnant women using EDTA anticoagulant tubes and stored them at 4°C. The blood sample was treated as follows: centrifuged at 4°C, 1600 g for 10 min and the plasma was collected carefully and dispensed into 2.0 ml Eppendorf tubes. The plasma was centrifuged again at 4°C, 16,000 g for another 10 min. The upper plasma was carefully divided into new 2.0 ml Eppendorf tubes and each contained approximately 600 ml plasma, - 80 C refrigerator to save. Thereafter, plasma-free cell DNA (cfDNA) was extracted by magnetic bead extraction using a DNA extraction kit (BGI, Wuhan, China). The extracted DNA library was constructed using a fetal chromosome aneuploidy (T21/T18/T13) detection kit (BGI), and high-throughput sequencing (0.5×) was performed using the combinatorial probe-anchor synthesis-based BGISEQ-500 platform (BGI). We mainly analyzed T21-, T18-, and T13-positive cases, along with an additional positivity analysis for SCAs, RATs, and MMS.

### Prenatal diagnosis by G-banded karyotyping

Amniocytes or cord blood cells were transferred to amniotic cell culture (Biosan, Zhejiang, China) and T cell culture media (Biosan), respectively, on an ultra-clean workbench for *in vitro* cell culture. When a specified number of cells were in the metaphase of active division, colchicine was added to inhibit mitosis. After the cells were digested by trypsin to isolate amniocytes, treated with hypotonic solution, fixed, and subjected to G-banded karyotyping, the karyotype was captured using an automatic scanner (Leica Microsystems, Wetzlar, Germany). We then manually counted 30 or more integrity cleavage phases and analyzed five or more for description according to the principles stated in *An International System for Human Cytogenetic Nomenclature, ISCN 2020*.

### Prenatal diagnosis by chromosomal microarray analysis

Amniocyte genomic DNA (250 ng) or umbilical cord blood cells was extracted using a QIAamp^®^ DNA Mini Kit (Qiagen, Hilden, Germany), after which it was digested, ligated, PCR-amplified, purified, fragmented, labeled, and hybridized to the Affymetrix CytoScan 750K array. The raw data were analyzed using the Chromosome Analysis Suite (ChAS) 4.2 (Affymetrix, Santa Clara, CA, United States). Interpretation and reporting of constitutional CNVs were performed according to the standards and guidelines released by the American College of Medical Genetics (Riggs et al., 2020). We described the clinical significance of CNVs under a five-tiered system: pathogenic, likely pathogenic, variants of uncertain significance, likely benign, and benign. In accordance with the aforementioned standards, we did not report microdeletions less than 500 kb, microduplications less than 1 Mb, and some CNVs with low penetrance (Rosenfeld et al., 2013; Armour et al., 2018). In addition, regions of homozygosity (ROH) with a size of more than 10 Mb were reported.

### Prenatal diagnosis by CNV-seq

Genomic DNA was extracted from amniocytes or umbilical cord blood cells using a Qiagen DNeasy Blood & Tissue Kit (Qiagen). Genomic DNA (50 ng) was prepared as a template to construct a sequencing library and sequenced using a NextSeq CN500 System (Illumina, San Diego, CA, United States). The sequencing results were subjected to bioinformatics analysis and annotated by the chromosome aneuploidy and gene microdeletion analysis software (Berry, Inc., Beijing, China). The whole experiment process was commissioned by Berry, Inc. The clinical evaluation of results showing CNVs (>100 kb) was based on the aforementioned guidelines.

### Statistical analysis

The positive predictive value (PPV) was calculated as the number of cases for which NIPS screening and confirmatory diagnostic testing were concordant (including mosaicism) divided by the number of cases with IPD results multiplied by 100. SPSS 26.0 software (SPSS Inc., Chicago, IL, United States) was used to determine the confidence interval of PPV.

## Results

### Positive predictive value of non-invasive prenatal screening

Within the study period, 53,437 pregnant women underwent NIPS examination at our institute, and 626 received positive results, with an overall positive rate of 1.17%. Among the 626 positive cases recorded at Changsha Hospital for Maternal and Child Health Care from January 2017 to June 2021, 59 patients refused prenatal genetic diagnosis, while 567 patients underwent IPD, with a diagnostic rate of 90.58%, which included 115 confirmed cases of common autosomal trisomies, 104 of SCAs, 18 of RATs, and 25 of MMS (Table 2). Moreover, the PPV for T21, T18, T13, SCAs, RATs, and MMS was 81.13% (86/106), 37.93% (22/58), 18.42% (7/38), 48.83% (104/213), 18.37% (18/98), and 41.67% (25/60), respectively. In addition, among the different types of SCAs, 47, XXY had the highest PPV (40/49, 81.63%); followed by 47, XYY (22/30, 73.34%); 47, XXX (23/44, 52.27%), and 45, X (19/90, 21.11%).

**TABLE 2:** Performance of NIPS in detecting trisomies and MMS in the 626 positive samples

Type of abnormalities		NIPS (*n*)	Prenatal diagnosis (*n*)		Diagnostic Rate (%)	With diagnosis results		PPV [% (95% CI)]
			Accepted (*n*)	Refused (*n*)		Accordance (*n*)	Discordance (*n*)	
Common autosomal trisomies	T21	110	106	4	96.36	86	20	81.13 (73.6–88.7)
	T18	59	58	1	98.31	22	36	37.93 (25.1–50.8)
	T13	40	38	2	95.00	7	31	18.42 (5.5–31.3)
SCAs	45, X	95	90	5	94.74	19	71	21.11 (12.5–29.7)
	47, XXY	54	49	5	90.74	40	9	81.63 (70.4–92.9)
	47, XXX	51	44	7	86.27	23	21	52.27 (36.9–67.6)
	47, XYY	38	30	8	78.95	22	8	73.34 (56.5–90.1)
RATs		114	98	16	85.96	18	80	18.37 (10.6–26.2)
MMS		71	60	11	84.51	25	35	41.67 (28.8–54.5)
Total		632*	573*	59	90.57	262	311	45.28 (41.6–49.8)

CI, confidence interval; ∗, Six cases suggested abnormalities on two chromosomes. Therefore, 6 more than the total of 626 and 567.

### Discordance between non-invasive prenatal screening and positive invasive prenatal diagnosis results

Among the 567 NIPS-positive samples, 48 cases were discordant with the positive IPD results except for cases of balanced structural rearrangement. We divided these cases into the following four categories according to the number of chromosomes considered for the evaluation based on NIPS and IPD (Table 3): 1) Multiple-to-one: NIPS results suggested multiple chromosome abnormalities, whereas IPD identified abnormality on only one of those chromosomes; 2) One-to-one: NIPS results suggested abnormality of one chromosome; IPD results also suggested abnormality of the same chromosome but were discordant with the NIPS result in terms of the location/type of the chromosomal aberration. This included mosaicism in 21 cases, partial deletion/duplication in 8 cases, and from monosomy to trisomy in 3 cases; 3) One-to-multiple: NIPS results suggested abnormality of one chromosome, whereas IPD results revealed multiple chromosome abnormalities that included the target chromosome; 4) One-to-another one: NIPS results suggested abnormality of one chromosome, whereas IPD identified the abnormality on another chromosome. In types “one-to-multiple” and “one-to-another one,” IPD reported several additional findings involving other chromosomes compared with those of NIPS, which included trisomy/partial trisomy, microdeletions/microduplications, and unbalanced structural rearrangements. Details are shown in Supplementary Table S1.

**TABLE 3:** Cases showing discordance between NIPS and positive IPD results.

NO.	Categories	NIPS results	Diagnosis results		Cases (*n*)	Total (*n*)
			Primary classification	Secondary classification		
1	Multiple-to-one	Abnormality of multiple chromosomes	Abnormality only on one of those chromosomes	Trisomy (*n* = 3)	3	
				Mosaicism (*n* = 21)		
2	One-to-one	Abnormality of one chromosome	Abnormality of the same chromosome	Partial deletion or duplication (*n* = 8)	32	
				From monosomy to trisomy (*n* = 3)		
3	One-to-multiple	Abnormality of one chromosome	Multiple chromosomal abnormalities that included the target chromosome	Trisomy of two or more (*n* = 2)	9	48
				Trisomy + sSMC (*n* = 1)		
				Unbalanced structural rearrangement (*n* = 6)		
4	One-to-another one	Abnormality of one chromosome	Abnormality on another chromosome	Trisomy of another (*n* = 1)	4	
				Microdeletion (*n* = 3)		

sSMC, small supernumerary marker chromosomes.

### Discordance between results of karyotyping and CMA/CNV-seq

Among the pregnant women who chose to proceed with the diagnostic procedures, 512 cases were diagnosed at our prenatal diagnosis center; 308 pregnant women opted for both karyotyping and CMA/CNV-seq. Discordant results between karyotyping and CMA/CNV-seq were found in 43 cases, accounting for 13.96% of the study population (Table 4 and Supplementary Table S2). This excluded chromosome polymorphisms, such as inv (9)(p12q13), inv (1)(p13q21), and inv(Y)(p11.2q11.2); seven discordant cases were associated with mosaicism, including four cases of sex chromosome mosaicism and three cases of autosomal mosaicism. Among these, six cases were successfully detected by karyotyping but not by CMV/CNV-seq, and for Case 304, while a normal karyotype was observed, the CMA result was arr (2) × 3 [0.52] hmz. Case 108 showed positive results for both karyotyping and CMA, with the CMA identifying the source of the small supernumerary marker chromosomes (sSMCs) detected by karyotyping. Karyotyping detected reciprocal translocation and inversion in cases 140 and 437, respectively; however, these balanced chromosome rearrangements were not identified by CMA. Moreover, 10 cases with MMS and 6 with ROH were detected by CMA in 193 samples with normal karyotypes, thus having improved diagnostic rates of 5.18% and 3.11%, respectively, compared with those for karyotyping. In addition, chromosome breakpoints in 17 cases with unbalanced rearrangements were detected relatively accurately by CMA/CNV-seq (Supplementary Table S2).

**TABLE 4:** Cases showing discordance between karyotyping and CMA/CNV-seq results.

No	Case number	Maternal Age (Years Old)	Gestational Age (Weeks *)	NIPS	Karyotype	CMA/CNV-seq results	Size (Mb)	Ultrasound findings	Pregnancy outcome
1	Case 309	30	16^+5^	XO	45, X [6]/46, XX [75]	N	—	*N*	Born
2	Case 312	27	18^+1^	XO	45, X [41]/47, XXX [20]	N	—	Single umbilical artery	TOP
3	Case 353	29	19^+1^	XO	45, X [6]/46, XX [84]	N	—	N	TOP
4	Case 493	28	16^+3^	XO	47, XXX [18]/46, XX [37]	N	—	N	Born
5	Case 122	37	20^+^	T13,	47, XN, +20 [28]/46, XN [22]	N	—	N	TOP
				T20					
6	Case 386	25	14^+3^	T4	47, XX, +4 [19]/46, XX [71]	N	—	N	TOP
7	Case 304	31	17^+3^	T2	N	arr (2)x3 [0.52] hmz	—	FGR, Oligohydramnios	TOP
8	Case 108	28	18^+^	T16	47, XN, +mar [14]/46, XY [18]	arr [GRCh37] 16p11.2q22.1 (33,766,659_67,589,639)x3 [0.52]	33.8	—	TOP
9	Case 140	48	20^+^	T16	46, XY, t (4;9) (q12;q22)[9]/46, XY [31]	N	—	N	Born
10	Case 437	33	20^+1^	XXX	46, XX, inv (6) (p21q13) mat	N	—	N	Born
11	Case 109	28	17^+^	MMS	N	arr [GRCh37] 5p15.33 (113,576_2,835,831)x1	2.7	N	TOP
12	Case 121	36	22^+^	MMS	N	arr [GRCh37] 3q23q25.31 (141158071_155492129)x3	14.3	N	TOP
13	Case 123	24	27^+^	MMS	N	arr [GRCh37] 2q24.1q31.1 (158448403_174291185)x1 dn	15.8	NT was 3.3 mm at 12 gestational age	TOP
14	Case 172	33	19^+^	MMS	N	arr [GRCh37] 16p13.11p12.3 (15319277_18172468)x1	2.8	N	Born
15	Case 242	30	17^+3^	T16	N	arr [GRCh37] 16p13.11p12.3 (15325072_18242713)x3 mat	2.9	N	Born
16	Case 347	31	17^+3^	T15	N	arr [GRCh37] 1p36.33 (849,466_1996635)x1 dn	1.15	Fetal tetralogy of Fallot, PLSVC, Thoracic vertebral abnormality	TOP
17	Case 64	28	26^+^	T21	N	arr [GRCh37] 13q33.3q34 (107382604_115107733)x1	7.7	FGR	TOP
18	Case 376	28	18^+4^	MMS	N	arr [GRCh37] 15q13.1q13.3 (28635057_32444261)x1 mat	3.81	N	Born
19	Case 164	27	20^+^	T15	N	arr [GRCh37] 15q11.2q13.1 (23281885_28526905)x4	5.2	N	TOP
20	Case 500	33	19^+4^	MMS	N	arr [GRCh37] 22q13.33 (50207711_51197766)x1	0.99	Normal indicators at 12 weeks	TOP
21	Case 86	38	18^+^	MMS	N	arr [GRCh37] 5p15.33p15.1 (113,576_16203210)x2 hmz	16.0	Missed follow-up	
22	Case 146	36	19^+^	MMS	N	arr [GRCh37] 2q31.1q37.3 (174605494_242773583)x2 hmz	68.1	FGR, Placental thickening,	TOP
								Oligohydramnios	
23	Case 156	31	18^+^	T16	N	arr [GRCh37] 16p13.3p12.3 (94,807_17705580)x2 hmz,	17.6,	N	Born
						16q22.3q24.3 (73772289_90146366)x2 hmz	16.3		
24	Case 240	32	20^+^	T13	N	arr [GRCh37] 18p11.23q12.2 (7131233_34755544)x2 hmz	27.6	N	Born
25	Case 477	27	16^+5^	MMS	N	arr [GRCh37] 18q21.32q23 (56947979_77997606) hmz	21.05	N	Born
26	Case 552	30	16^+4^	CNV	N	arr [GRCh37] 18p11.32p11.21 (136,305_11807701)x2 hmz	11.67	N	Born

XO, 45, X high risk; XXX, 47, XXX high risk; N, Normal;/: No; PLSVC, persistent left superior vena cava; NT, nuchal translucency; TOP, termination of pregnancy; ∗, weeks + days.

### Analysis of pregnancy follow-up

**FIGURE 1:**
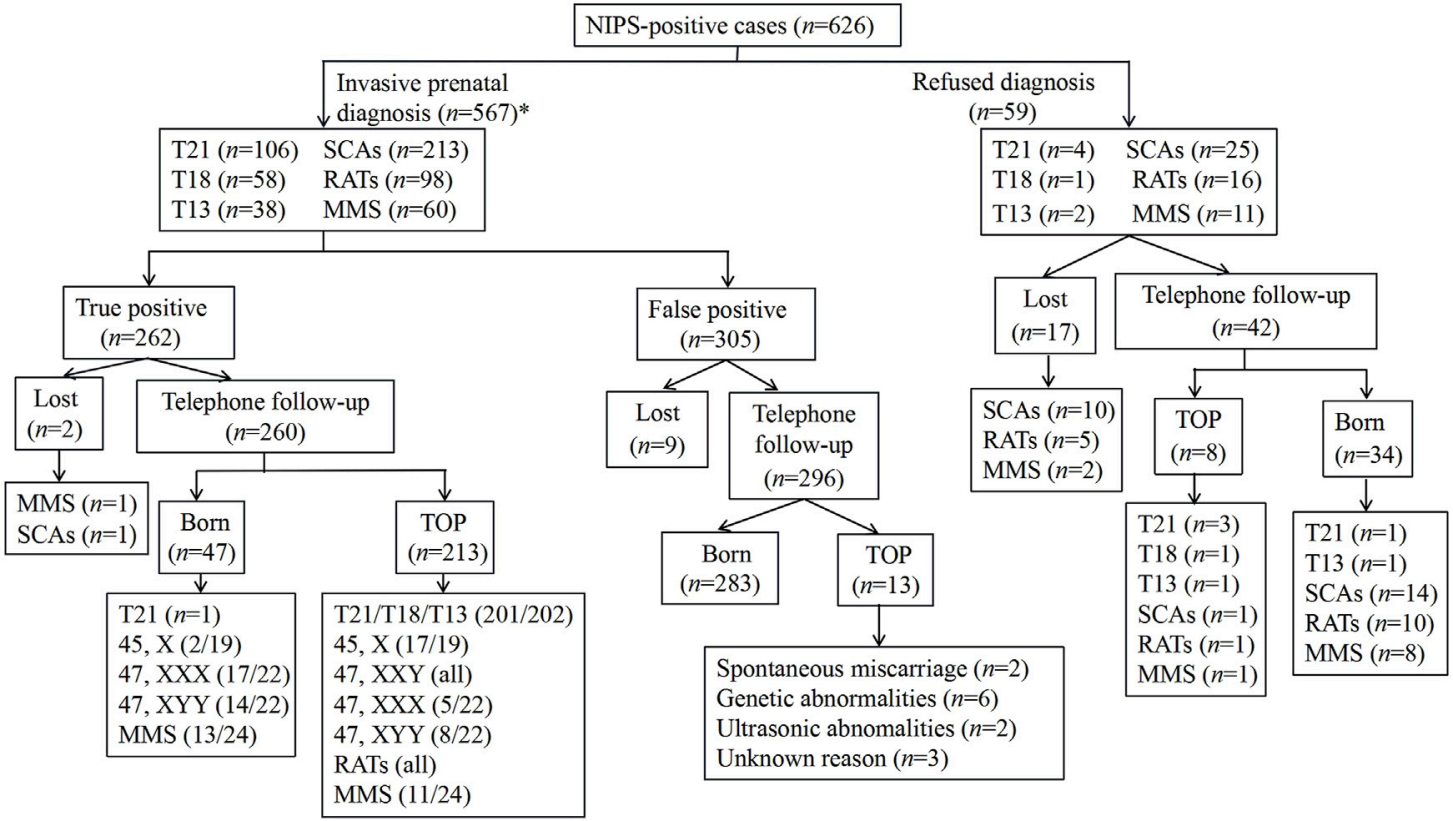
Outcomes of all NIPS-positive cases.TOP, termination of pregnancy; *, six cases suggested abnormalities on two chromosomes. Therefore, the sum in the box is six more than 567.

We followed up on all the NIPS-positive cases (Figure 1). Among the 567 pregnant women who underwent IPD, 262 were confirmed as true positive cases. Tracking the pregnancy outcomes of 260 pregnant women among them led to the following observations: mothers of all fetuses diagnosed with T21, T13, T18, RATs, Klinefelter syndrome, and Turner syndrome terminated their pregnancies, excluding one T21 case (Supplementary Table S1; Case 439) and two cases of haploid chromosome X with a low rate of mosaicism and normal ultrasound findings throughout pregnancy (Supplementary Table S1; cases 309 and 512); five cases diagnosed as having fetuses with Triple X syndrome and eight cases diagnosed as having fetuses with 47, XYY syndrome terminated their pregnancies, with birth rates of 77.27% (17/22) and 63.64% (14/22), respectively. Among the MMS cases detected by NIPS, the clinical significance of most cases was unknown, and due to the presence of pathogenic CNVs, only 45.83% (11/24) cases terminated their pregnancies. Additionally, among the 305 cases confirmed to be false positives, pregnancy outcome tracking of 296 pregnant women showed the following: two patients underwent spontaneous abortion; six patients terminated their pregnancies due to other genetic abnormalities; two patients had abortions due to abnormal ultrasound findings; three patients terminated their pregnancies for unknown reasons, and the remaining 283 mothers had infants that were born healthy.

Among the 59 pregnant women who refused prenatal genetic diagnosis, the pregnancy outcomes of 42 women were tracked: eight patients terminated their pregnancies due to multiple malformations found by ultrasound, and 34 underwent delivery. Among the cases that resulted in live births, a confirmed occurrence of T21 was found in an infant from a twin pregnancy, and the remaining 33, which included one case of T13, reported healthy births that were confirmed by long-term follow-up.

## Discussion

From 2012 onwards, NIPS for fetal aneuploidies has been broadly implemented for detecting common autosomal trisomies and SCAs owing to the advantages associated with it, such as non-invasiveness, zero risk for the unborn baby, capability to acquire diagnostic hints as early as the 10th week of gestation onwards, immediate results within as early as 2 weeks, as well as high sensitivity (99.3% for T21, 97.4% for T18, and 97.4% for T13) and specificity (pooled specificity was 99.9% for all three trisomies) (Taylor-Phillips et al., 2016; Liehr, 2021). However, this approach identifies only 75%–85% of clinically relevant aneuploidies (Pescia et al., 2017). Therefore, additional screening based on identifying RATs and MMS is necessary. Here, we assessed a series of 626 NIPS-positive cases with low genomic coverage and detected a broad range of aneuploidy classes, namely the common autosomal trisomies, SCAs, RATs, and MMS. The PPV of T21 (81.23%) observed using our platform in the present study was within the range of values reported in published literature (between 80% and 90%) (Junhui et al., 2021). The PPVs of T18 and T13, presented as the main positive results, were 37.93 and 18.42%, respectively, slightly lower than those reported by previous studies using the same platform (Lu et al., 2020). The PPVs of SCAs, RATs, and MMS, presented as additional positive results, were 48.83, 18.37, and 41.68%, respectively, slightly higher than those reported by previous studies using the same platform (Wang et al., 2021). PPVs obtained via NIPS, excluding that of T21, are known to have large variations associated with prior risk factors, such as maternal age and individual trisomies (Petersen et al., 2017; Skrzypek and Hui, 2017). NIPS results are affected by an insufficient or absent fetal fraction, fetoplacental mosaicism, the presence of a vanishing twin, maternal mosaicism, maternal CNVs, and maternal malignancy, leading to false positives that are discordant with results obtained by other methods (Hartwig et al., 2017; Samura and Okamoto, 2020). Moreover, technical factors such as testing procedures, sequencing algorithms, and depths, as well as Z-scores, may also be important in terms of their effect on NIPS results (Junhui et al., 2021). This makes the fluctuation of the PPV of NIPS in different study populations a common occurrence. In our research, we found that RATs have a PPV of 18.37%, similar to that of T13 presented as the main positive results and could therefore act as an extension of NIPS screening. MMS had a higher PPV than that of T18 presented as positive results, but most of the CNVs were identified as hereditary and of unknown significance. Disclosure of these results to pregnant women did not provide them any substantial help with pregnancy-related decisions and had a negative psychological impact on them. Therefore, for cases of MMS suggested by NIPS results, it is recommended to only present the diagnoses to pregnant women if the CNVs are in genomic regions that have definite associations with certain syndromes or after pathogenicity has been identified.

Discordant results associated with NIPS often occur during screening and diagnosis. At present, the discordant cases reported in literature mainly focus on false positive and false negative NIPS cases ^[26]^. In this research, we focused on true positive cases and identified 48 discordant cases (which accounted for 8.47% of the total cases) between the positive results of NIPS and IPD. Assessment of the cases in our analysis confirmed the importance of testing by IPD in addition to NIPS. There are four main reasons for the discordance. First, there was a certain degree of false positivity in NIPS, so it can not accurately determine abnormalities as being on one or two chromosomes in type of “Multiple-to-one”. Second, NIPS has high detection rates coupled with high sensitivity for common fetal aneuploidies (trisomies 13, 18, and 21), but the screening accuracy for SCAs, RATs, and CNVs is lower than that for the common autosomal aneuploidies (Taylor-Phillips et al., 2016; Liehr, 2021; Wang et al., 2021). Therefore, some aneuploidies and CNVs have been found unexpectedly in IPD. Third, it must be considered that NIPS, which is based on second-generation sequencing technologies, is not sensitive to some DNA fragments with a high average content of guanine and cytosine bases, and the sequencing depth of NIPS also means that NIPS cannot give more genetic information about some sSMCs and CNVs (Ye et al., 2021). Last, NIPS is cffDNA-based non-invasive prenatal screening. In pregnant women, the small amount of plasma cffDNA is believed to be a contribution from the cytotrophoblast cells of the chorionic villi in the placenta (Lun et al., 2008). NIPS identifies fetal genetic abnormalities under the assumption that the cytogenetic constitution of the placenta matches that of the fetus. However, during embryonic development, mitotic error and trisomy, monosomy, and deletion rescue can lead to two (or more) genetically different cell lines that differentiate into different parts. As a result, the karyotype of cytotrophoblast cells does not always represent fetal chromosome constitution (Van Opstal and Srebniak, 2016). Besides, the different occurrence times of mitotic non-disjunction of different chromosomes in early embryo development results in varying levels of chromosomal mosaicism in different placental and fetal tissues. Among our discordant cases, we found that NIPS suggested trisomy/monosomy in 21 cases where IPD results indicated mosaicism. This accounted for the largest proportion of discordance observed between NIPS and IPD results. Our observations show that in some cases diagnosed with very low rates of mosaicism confirmed by multiple detection methods, pregnant women choosing to continue with pregnancy had fetuses that developed well after birth (Supplementary Table S1; Case 439). Therefore, it is advised that pregnant women who receive positive NIPS results should not hasten to adopt a negative attitude and should actively undergo follow-up consultations to identify the abnormality by means of IPD; only then can they make decisions regarding the continuation or termination of pregnancy. Accordingly, NIPS should not be regarded as a diagnostic tool for conclusive diagnoses, and positive NIPS results must be further assessed using invasive prenatal genetic diagnostic and ultrasonic diagnostic approaches.

G-banded karyotyping, which has limited resolution (5–10 Mb), is a common diagnostic technique and the gold standard for diagnosing chromosomal disorders. It can detect chromosomal aneuploidy or polyploidy, large chromosomal deletions/duplications, and balanced chromosomal rearrangement. Other commonly used prenatal diagnostic techniques, namely CMA and CNV-seq, can be used to analyze aneuploidy as well as microdeletion and microduplication (≥100 kB) (Armour et al., 2018; Zhao and Fu, 2019). In our study, 43 discordant cases were found in the chromosomal analysis of 308 patients performed using karyotyping and CMA/CNVseq. Four instances of sex chromosome mosaicism were detected by karyotyping but not by CMA. For cases of sex chromosome abnormality indicated by NIPS, karyotyping was seen to be more effective than CMA in confirming true positive detection of sex chromosome mosaicism. Additionally, two cases of autosomal mosaicism were detected by karyotyping but not by CMA, whereas one case of autosomal mosaicism was detected by CMA but not by karyotyping.

Karyotyping and CMA each have certain advantages and disadvantages for their use in detecting autosomal mosaicism. Although karyotyping requires cell culture, it can detect mosaics of different types, including those of a very low proportion. However, multiple factors, such as aberration of the primary amniotic cells themselves and cell aberration resulting from *in vitro* culturing, may lead to pseudomosaicism, a loss or increase in the abnormal cell line resulting in a change in the proportion of mosaic cells, or even to missing the detection of autosomal mosaicism (Fan et al., 2021). Conversely, CMA can only stably detect mosaicism in cells with larger proportions (>30%) of it and can directly detect the amniotic fluid genome, thus being capable of reflecting the proportion of true mosaicism in the sample. Additionally, CMA has the unique advantage of being able to detect CNVs and ROH, which cannot be detected by karyotyping. Our results show that compared with CNVs detected by karyotyping, 10 more cases of pathogenic CNVs were detected by CMA, indicating an improved diagnostic rate of 5.18% compared with that of karyotyping. In addition, for NIPS-positive samples showing normal karyotypes, a total of 3.11% ROH was detected by SNP-based microarrays. The presence of large fragments of ROH in the fetus is associated with the risk of uniparental disomy (UPD), which is the result of the successful rescue of cells from aneuploidy to euploidy after germ cell fertilization. A UPD diagnosis should be considered when NIPS suggests trisomy, especially on chromosomes 6, 7, 11, 14, 15, and 20 (Benn, 2021). Thus, it can be seen that a single detection method can easily lead to misdiagnosis. Therefore, combining karyotyping with CMA seems preferable for obtaining accurate diagnoses of chromosomal abnormalities.

At the later stages of follow-up, most women with fetuses diagnosed with autosomal trisomies had terminated pregnancy, excluding one case of T21 with a low rate of mosaicism. SCAs are the most frequent chromosomal abnormalities encountered in NIPS. In true positive cases, the overall termination of pregnancy rate was 22.7% (5/22) for Triple X syndrome and 36.36% (8/22) for 47, XYY syndrome, which was significantly lower than those for other chromosomal syndromes. The prevalence of Triple X and 47, XYY syndromes among newborns is high at 11 per 100,000 females and 18 per 100,000 males, respectively (Gruchy et al., 2016). Although an increased risk of psychosocial problems or psychiatric disorders (such as autism) during childhood has been associated with the 47, XYY syndrome, long-term, unbiased follow-up studies have concluded that Triple X and 47, XYY syndromes do not cause postnatal development disorders. Children with these conditions have IQs in the normal range despite physical abnormalities being occasionally observed (Berglund et al., 2019). The acceptance of fetuses with SCAs tends to be affected by many factors, such as social and cultural background, disease type, genetic counseling methods, and the economic status of the family. In China, an increasing number of people are accepting children with Triple X and 47, XYY syndromes. Therefore, the exclusion of Triple X and 47, XYY syndromes from the NIPS process is expected in the near future. Moreover, the true or false positive nature of ultrasound findings is also an important factor in determining the decision to continue a pregnancy.

## Conclusion

NIPS has a high positive rate for detecting common trisomies and SCAs in the general testing of pregnant women, and testing for RATs and MMS can be additionally conducted with the informed consent of pregnant women to obtain a more accurate diagnosis. However, NIPS cannot be used as a substitute for amniocentesis and prenatal diagnosis techniques owing to its high rate of false positives and discordance with diagnoses provided by IPD. CMA combined with karyotyping can be recommended as the preferred method of prenatal diagnosis for cases where NIPS results indicate a high risk in pregnancy.

## Data Availability

The original contributions presented in the study are included in the article/Supplementary Material, further inquiries can be directed to the corresponding author.
