# Supplementary material for: A retrospective single-center analysis of prenatal diagnosis and follow-up of 626 chinese patients with positive non-invasive prenatal screening results
Source: Front Genet. 2022 Sep 19;13:965106. doi: 10.3389/fgene.2022.965106 (PMC9527272; doi:10.3389/fgene.2022.965106)
Supplement: Supplementary file 1 [file Table1.doc]

Supplementary Table S1. Detailed cases showing discordance between NIPS and positive IPD results

| NO. | Primary  Classification | Secondary classification | Case number | Age | Gestational age (week) | NIPS results | Karyotype | CMA/CNV-seq results | Pregnancy outcome |
| --- | --- | --- | --- | --- | --- | --- | --- | --- | --- |
| 1 | Multiple-to-one |  | Case 5 | 35 | 17^+^ | T21 and XYY | 47,XYY | / | Born |
| 2 |  |  | Case 122 | 37 | 20^+^ | T13 and T20 | 47,XN, +20[28]/46, XN[22] | N | TOP |
| 3 |  |  | Case 356 | 31 | 15^+4^ | T18 and XXX | 47,XXX | / | TOP |
| 4 | One-to-one | Mosaicism | Case 66 | 25 | 19^+^ | T14 | 47, XN, +14[8]/46, XY[51] | / | TOP |
| 5 |  |  | Case 70 | 21 | 24^+^ | XO | 45, X[44]/46, X, del(X)(q24)[13] | / | TOP |
| 6 |  |  | Case 81 | 36 | 17^+^ | T8 | 47, XX, +8[3]/46, XX[57] | / | TOP |
| 7 |  |  | Case 91 | 32 | 20^+^ | T13 | 47, XN, +13[27]/46, XX[20] | / | TOP |
| 8 |  |  | Case 115 | 35 | 21^+^ | XO | 45, X[49]/46, XX[13] | / | TOP |
| 9 |  |  | Case 197 | 31 | 14^+3^ | XO | 45, X/46, XX mosaic | / | TOP |
| 10 |  |  | Case 225 | 28 | 17^+2^ | T21 | 47, XY, +21[8]/46, XY[46] | arr(21)x3[0.23] | TOP |
| 11 |  |  | Case 230 | 23 | 16^+6^ | XO | 45, X[19]/47, XXX[1]/46, XX[62] | arr(X)x1[0.21] | TOP |
| 12 |  |  | Case 248 | 25 | 16^+1^ | XO | 45, X[10]/46, XX[50] | arr(X)x1[0.27] | TOP |
| 13 |  |  | Case 309 | 30 | 16^+5^ | XO | 45, X[6]/46, XX[75] | N | Born |
| 14 |  |  | Case 312 | 27 | 18^+1^ | XO | 45, X[41]/47, XXX[20] | N | TOP |
| 15 |  |  | Case 333 | 31 | 12^+5^ | XO | 45, X[17]/46, XX[48] | arr[GRCh37] (X)x1[0.5], 22q11.21(18970561_21800471)x3 | Spontaneous abortion |
| 16 |  |  | Case 345 | 32 | 14^+6^ | XO | 47, X, i(X)(q10)X2[11]/45, X[9]/46, XX[50] | arr[GRCh37] Xp22.33q11.1(168,551-62,061,892)x1[0.35] | TOP |
| 17 |  |  | Case 353 | 29 | 19^+1^ | XO | 45, X[6]/46, XX[84] | N | TOP |
| 18 |  |  | Case 359 | 28 | 17^+3^ | T16 | 47, XY, +16[5]/46, XY[75] | arr(16)x3[0.26] | TOP |
| 19 |  |  | Case 379 | 41 | 14^+6^ | XYY | 46, XY/47, XYY mosaic | / | Born |
| 20 |  |  | Case 439 | 38 | 17^+6^ | T21 | 47, XX, +21[3]/46, XX[71] | arr[GRCh37] 21q11.2q22.3(15016486_48093361)x3[0.23] | Born |
| 21 |  |  | Case 463 | 27 | 19^+6^ | T2 | 47, XY, +2[3]/46, XY[98] | arr(2)x3[0.33] |  |
| 22 |  |  | Case 466 | 26 | 19^+4^ | XO | 45 ,X[26]/46, XX[54] | arr(X)x1[0.39] |  |
| 23 |  |  | Case 491 | 25 | 17^+3^ | XO | 45, X[11]/46, XX[81] | arr(X)x1[0.15] | TOP |
| 24 |  |  | Case 512 | 24 | 19^+^ | XO | 45, X[4]/46, XX[46] | arr(X)x1[0.34] | Born |
| 25 |  | Partial deletion or duplication | Case 12 | 29 | 17^+^ | T10 | 46, XX?dup(10)(q25.1q25.31) | / | TOP |
| 26 |  |  | Case 22 | 28 | 18^+^ | T9 | 46, XX?dup(9)(q21q22) | / | Lost follow-up |
| 27 |  |  | Case 60 | 31 | 22^+^ | Monosomy 7 | 46, XY, del(7)(q35) | / | TOP |
| 28 |  |  | Case 83 | 20 | 18^+^ | T13 | 46, XX, ins(13)(q34q21q34) | arr[GRCh37] 13q21.2q34(59879188_112632036)x3,13q34(112632255_115107733)x1 | TOP |
| 29 |  |  | Case 108 | 28 | 18^+^ | T16 | 47, XN, +mar[14]/46, XY[18] | arr[GRCh37] 16p11.2q22.1(33766659_67589639)x3[0.52] | TOP |
| 30 |  |  | Case 135 | 36 | 19^+^ | XXX | 46, X, i(X)(q10;q10) | / | TOP |
| 31 |  |  | Case 330 | 32 | 17^+1^ | XO | 46, X, del(X)(q21.3) | arr[GRCh37] Xp22.33q21.31(168807_90824100)x1[0.25], Xq21.31q28(90934301_155233098)x1 | TOP |
| 32 |  |  | Case 511 | 30 | 17^+5^ | XO | 46, X, i(Xq) | arr[GRCh37] Xp22.33p21.2(168551_30580694)x1,Xq21.1q28(81697155_155233098)x3 | TOP |
| 33 |  | From monomy to Trisomy | Case 352 | 27 | 18^+3^ | XO | 47,XXX | arr(X)x3 | TOP |
| 34 |  |  | Case 430 | 39 | 19^+6^ | XO | 47,XXX | arr(X)x3 | Born |
| 35 |  |  | Case 493 | 28 | 16^+3^ | XO | 47, XXX[18]/46, XX[37] | N | Born |
| 36 | One-to-multiple | Trisomy of two or more | Case 85 | 29 | 17^+^ | T18 | 48, XYY, +18 | / | TOP |
| 37 |  |  | Case 246 | 34 | 16^+2^ | XXY | 47, XXY[66]/48, XXY, +20[8] | / | TOP |
| 38 |  | Trisomy + sSMC | Case 144 | 23 | 23^+^ | T21 | 48, X, inv(Y)(p11q11), +21, +mar | / | TOP |
| 39 |  | Unbalanced rearrangement | Case 57 | 32 | 17^+^ | T7 | 46, XY, der(9)t(7;9)(q31;p22) | / | TOP |
| 40 |  |  | Case 173 | 31 | 18^+^ | dup(7p22.3p21.3,  13.2 Mb) | 46, XY, der(8)?t(7;8)(p21.3;p23.3) | arr[GRCh37] 7p22.3p21.3(43376_12705586)x3,  8p23.3(158048_1569968)x1 | TOP |
| 41 |  |  | Case 260 | 28 | 17^+1^ | dup(16q22.1q24.3,  21.59 Mb) | 46, X, der(X)t(X;16)(q28;q22) | arr[GRCh37] Xq28(154553185_155233098)x1,  16q22.1q24.3(68617218_90155062)x3 | TOP |
| 42 |  |  | Case 265 | 29 | 16^+4^ | T18 | 45, XY, der(13;14)(q10;q10), del(18)(p11.21) | arr[GRCh37] 18p11.22p11.21(9308617_15143715)x1,18p11.31p11.22(3477777_9200810)x1 | TOP |
| 43 |  |  | Case 284 | 29 | 16^+6^ | dup(10q25.2-q26.3,  21.59 Mb) | 46, XX, der(1)t(1;10)(p36.3;25.2) | arr[GRCh37] 10q25.2q26.3(113846815_135426386)x3 |  |
| 44 |  |  | Case 438 | 27 | 19^+1^ | XYY | 46, X, der(X)t(X;Y)(q23;q11.2) | arr[GRCh37] Xq23q28(111151173_155233098)x1，Yq11.221q11.23(15761679_28799654)x1 | TOP |
| 45 | One-to-another one | Trisomy of another | Case 25 | 29 | 21^+^ | T21 | 47, XXX | / | TOP |
| 46 |  | Microdeletion | Case 64 | 28 | 26+ | T21 | 46,XY | arr[GRCh37] 13q33.3q34(107382604_115107733)x1 | TOP |
| 47 |  |  | Case 347 | 31 | 17^+3^ | T15 | 46,XX | arr[GRCh37] 1p36.33 (849466_1996635)x1 dn | TOP |
| 48 |  |  | Case 500 | 33 | 19^+4^ | T14 and dup(14q23.3-q32.33,41.41 Mb) | 46,XX | arr[GRCh37] 22q13.33(50207711_51197766)x1 | TOP |

XO: 45,X high risk; XXX: 47,XXX high risk; XXY: 47,XXY high risk; XYY: 47,XYY high risk; dup: duplication; N: Normal; /: No; TOP: Termination of pregnancy
