# Supplementary material for: A retrospective single-center analysis of prenatal diagnosis and follow-up of 626 chinese patients with positive non-invasive prenatal screening results
Source: Front Genet. 2022 Sep 19;13:965106. doi: 10.3389/fgene.2022.965106 (PMC9527272; doi:10.3389/fgene.2022.965106)
Supplement: Supplementary file 2 [file Table2.doc]

Supplementary Table S2. Cases with unbalanced rearrangements detected by karyotyping and CMA/CNV-seq

| NO. | Case number | Age(year) | Gestatio-nal age (week) | NIPS results | Karyotype | CMA/CNV-seq results | Size  (Mb) | Pregnan-cy outcome |
| --- | --- | --- | --- | --- | --- | --- | --- | --- |
| 1 | Case 83 | 20 | 18^+^ | T13 | 46, XX, ins(13)(q34q21q34) | arr[GRCh37] 13q21.2q34(59879188_112632036)x3, 13q34(112632255_115107733)x1 | 52.75,  2.48 | TOP |
| 2 | Case 173 | 31 | 18^+^ | dup(7p) | 46, XY, der(8)?t(7;8)(p21.3;p23.3) | arr[GRCh37] 7p22.3p21.3(43376_12705586)x3,8p23.3(158048_1569968)x1 | 12.6,  1.41 | TOP |
| 3 | Case 260 | 28 | 17^+1^ | dup(16q22.1-q24.3,21.59 Mb) | 46, X, der(X)t(X;16)(q28;q22) | arr[GRCh37] Xq28(154553185_155233098)x1,  16q22.1q24.3(68617218_90155062)x3 | 0.68,  12.5 | TOP |
| 4 | Case 265 | 29 | 16^+4^ | T18 | 45, XY, der(13;14)(q10;q10), del(18)(p11.21) | arr[GRCh37] 18p11.22p11.21(9308617_15143715)x1, 18p11.31p11.22(3477777_9200810)x1 | 5.84,  5.72 | TOP |
| 5 | Case 266 | 29 | 16^+4^ | dup(9p24.2-p11.2,43.12M) | 47, XY, +der(9)del(9)(q11) | arr[GRCh37] 9p24.3p13.1(208454_38772005)x3 | 38.56 | TOP |
| 6 | Case 284 | 29 | 16^+6^ | dup(10q25.2-q26.3,21.59 Mb) | 46, XX, der(1)t(1;10)(p36.3;q25.2) | arr[GRCh37] 10q25.2q26.3(113,846,815-135,426,386)x3 | 21.58 | TOP |
| 7 | Case 330 | 32 | 17^+1^ | XO | 46, X, del(X)(q21.3) | arr[GRCh37] Xp22.33q21.31(168807_90824100)x1[0.25], Xq21.31q28(90934301_155233098)x1 | 90.6 | TOP |
| 8 | Case 331 | 23 | 15^+6^ | dup(13q14.3-q21.33,16.89 Mb) | 46, XX, dup(13)(q21.1q21.33) | arr[GRCh37] 9p24.3(1373386_2084563)x3,13q21.1q21.33(57911831_72164913)x3 | 0.71,  14.2 | TOP |
| 9 | Case 332 | 37 | 16^+6^ | del(13q33.1-q34,12.24 Mb) | 46, XX, del(13)(q33) | arr[GRCh37] 13q33.1q34(101866802_115107733)x1,3p26.3(61891_1833607)x3 | 13.24,  1.77 | TOP |
| 10 | Case 335 | 32 | 16^+^ | del(Xq22.3-q28,46.57 Mb) | 46, X, del(X)(q22) | arr[GRCh37] Xq23q28(109412332_155233098)x1 | 45.8 | TOP |
| 11 | Case 345 | 32 | 14^+6^ | XO | 47, X, i(X)(q10)X2[11]/  45, X[9]/46, XX[50] | arr[GRCh37] Xp22.33q11.1(168551_62061892)x1[0.35] | 61.9 | TOP |
| 12 | Case 367 | 32 | 17^+4^ | dup(9p24.3-p21.1,29.63 Mb)；del(13q33.3-q34,5.00 Mb) | 46, XY, 1qh+, der(13)t(9;13)(p13;q32) | arr[GRCh37] 9p24.3p21.1(208454_29653331)x3,13q33.3q34(109149281_115107733)x1 | 29.4,  5.9 | TOP |
| 13 | Case 382 | 22 | 15^+5^ | del(5q22.2-q23.2，13.36 Mb) | 46, XY, del(5)( q22.2q23.2) | arr[GRCh37] 5q22.2q23.2(112653239_126753687)x1 mat | 14.1 | TOP |
| 14 | Case 423 | 33 | 14^+^ | dup(7q31.33-q36.3,33.06M); del(18q21.32-q23,20.92 Mb) | 46, XX, der(18)t(7;18)(q31.3;q21.1) | arr[GRCh37] 7q31.33q36.3(126260825_159119707)x3, 18q21.32q23(56871832_78013728)x1 | 32.86,  12.14 | TOP |
| 15 | Case 438 | 27 | 19^+1^ | XXY | 46, X, der(X)t(X;Y)(q23;q11.2) | arr[GRCh37] Xp22.33q23(2703822_111150307)x2，Xq23q28(111151173_155233098)x1，Yp11.31q11.221(2650424_15757956)x0，Yq11.221q11.23(15761679_28799654)x1 | 108.45,  44.08,  13.11,  13.04 | Spontaneous abortion |
| 16 | Case 473 | 28 | 16^+6^ | del(5p15.31-p14.3,11.63 Mb) | 46, XX, del(5)(p14.2) | arr[GRCh37] 5p15.31p14.3(9482842_20319547)x1 | 10.84 | TOP |
| 17 | Case 561 | 28 | 16^+^ | dup(3q23-q25.1,10.16 Mb) | 46, XX, dup(3)(q25q23) | arr[GRCh37] 3q23q25.1(141440311_152054196)x4 | 10.61 | TOP |

XO: 45,X high risk; XXY: 47,XXY high risk; dup: duplication; N: Normal; /: No; TOP: Termination of pregnancy
